# Chitosan microparticles loaded with essential oils inhibit duo-biofilms of *Candida albicans* and *Streptococcus mutans*

**DOI:** 10.1590/1678-7757-2023-0146

**Published:** 2023-09-15

**Authors:** Lana Glerieide Silva GARCIA, Maria Gleiciane da ROCHA, FREIRE Rosemayre Souza, Paulo Iury Gomes NUNES, João Victor Serra NUNES, Mirele Rodrigues FERNANDES, Waldemiro Aquino PEREIRA-NETO, José Júlio Costa SIDRIM, Flavia Almeida SANTOS, Marcos Fábio Gadelha ROCHA, Lidiany Karla Azevedo RODRIGUES, Rodrigo Silveira VIEIRA, Raimunda Sâmia Nogueira BRILHANTE

**Affiliations:** 1 Universidade Federal do Ceará Departamento de Engenharia Química Ceará Brasil Universidade Federal do Ceará, Departamento de Engenharia Química, Ceará, Brasil.; 2 Universidade Estadual do Ceará Faculdade de Medicina Veterinária Ceará Brasil Universidade Estadual do Ceará, Faculdade de Medicina Veterinária, Ceará, Brasil.; 3 Universidade Federal do Ceará Departamento de Física, Central Analítica Ceará Brasil Universidade Federal do Ceará, Departamento de Física, Central Analítica, Ceará, Brasil.; 4 Universidade Federal do Ceará Faculdade de Medicina Departamento de Fisiologia e Farmacologia Ceará Brasil Universidade Federal do Ceará, Faculdade de Medicina, Departamento de Fisiologia e Farmacologia, Laboratório de Produtos Naturais, Ceará, Brasil.; 5 Universidade Federal do Ceará Faculdade de Medicina Departamento de Patologia e Medicina Legal Ceará Brasil Universidade Federal do Ceará, Faculdade de Medicina, Departamento de Patologia e Medicina Legal, Centro Especializado em Micologia Médica, Ceará, Brasil.; 6 Universidade Federal do Ceará Faculdade de Farmácia, Odontologia e Enfermagem Departamento de Odontologia Restauradora Ceará Brasil Universidade Federal do Ceará, Faculdade de Farmácia, Odontologia e Enfermagem, Departamento de Odontologia Restauradora, Ceará, Brasil.

**Keywords:** Drug effects on biofilms, Dental plaque, Candida albicans, Chitosan analogs & derivatives, Essential oils

## Abstract

Oral candidiasis is a common fungal infection that affects the oral mucosa, and happens when *Candida albican*s interacts with bacteria in the oral microbiota, such as *Streptococcus mutans*, causing severe early childhood caries. *C. albicans* and *S. mutans* mixed biofilms are challenging to treat with conventional antimicrobial therapies, thus, new anti-infective drugs are required.

**Objective:**

This study aimed to test a drug delivery system based on chitosan microparticles loaded with geranium and lemongrass essential oils to inhibit *C. albicans* and *S. mutan*s mixed biofilms.

**Methodology:**

Chitosan microparticles loaded with essential oils (CM-EOs) were obtained by spray-drying. Susceptibility of planktonic were performed according CLSI at 4 to 2,048 µg/mL. Mixed biofilms were incubated at 37ºC for 48 h and exposed to CM-EOs at 256 to 4,096 µg/mL. The antimicrobial effect was evaluated using the MTT assay, with biofilm architectural changes analyzed by scanning electron microscopy. RAW 264.7 cell was used to evaluate compound cytotoxicity.

**Results:**

CM-EOs had better planktonic activity against *C. albicans* than *S. mutans*. All samples reduced the metabolic activity of mixed *C. albicans* and *S. mutans* biofilms, with encapsulated oils showing better activity than raw chitosan or oils. The microparticles reduced the biofilm on the slides. The essential oils showed cytotoxic effects against RAW 264.7 cells, but encapsulation into chitosan microparticles decreased their toxicity.

**Conclusion:**

This study demonstrates that chitosan loaded with essential oils may provide an alternative method for treating diseases caused by *C. albicans* and *S. mutans* mixed biofilm, such as dental caries.

## Introduction

Oral candidiasis is a common fungal infection that affects the oral mucosa, and *Candida albicans* is the species most isolated from oral cavities and accounts for around 95% of cases^1^. Oral prostheses, host habits, hyposalivation, and other microorganisms in the oral cavity offer an ideal environment for *C. albicans* colonization.^2^ *C. albicans* can interact with other potential members of the oral bacterial microbiota, such as *Streptococcus mutans*^1^*. S. mutans* is one of the major contributors to oral diseases associated with biofilm presence because it can survive at low pH environments, producing substantial amounts of acid, leading to dental enamel demineralization and, therefore, dental caries.^3^ The association of *C. albicans* and *S. mutans* is often related to severe or recurrent early childhood caries (ECC) caused by dental biofilm presence^4^ The co-infection with *C. albicans* and *S. mutans* is more difficult to eradicate than their isolated infections, with the effective cariogenic characteristics of *Candida* spp. considerably influencing the development and progression of severe ECC.^4,5^

High resistance to antimicrobial therapies is a relevant clinical issue due to the increase of mixed biofilm-related infections.^6^ Various new anti-infective drugs have been developed with a broad action mode, effectively targeting prokaryotic and eukaryotic pathogenic biofilm cells. Natural polymers, such as chitosan, a linear polysaccharide obtained from partial deacetylation of chitin, have been used against different microorganism’s planktonic and biofilm cells.^7,8^ This biopolymer has been widely used as an antimicrobial agent due to its biocompatibility, biodegradability, low toxicity, and high functional potential imparted by its amino and hydroxyl groups.^9^ Different chitosan formulations showed activity against mono-species biofilms of *C. albicans* and *S. mutans*, with nanoparticles reducing biofilms in their mixed biofilm.^8,10,11^

Other natural products, such as essential oils, have antifungal activity against *C. albicans* in vulvovaginal infections. However, these compounds are highly volatile and toxic.^12^ Therefore, alternative strategies such as encapsulation have shown to be promising.^13^ Previously, chitosan microparticles loaded with geranium and lemongrass essential oils showed activity against *C. albicans* biofilms, reducing its biomass by up to ٨٠٪.^14^ The same 80% reduction was found in chitosan-coated catheters against mixed *S. epidermidis* and *C. albicans* biofilms.^15^

Given the potential of these compounds against mono and multispecies *Candida* biofilms, this study aims to test a drug delivery system based on chitosan microparticles loaded with geranium and lemongrass essential oils for inhibiting *C. albicans* and *S. mutans* mixed biofilms.

## Methodology

### Compounds

This study used previously characterized chitosan with low molecular weight (448869 - Sigma-Aldrich, San Luis, Missouri, USA).^16^ The chitosan’s molecular weight (MW) and deacetylation degree (DD) were determined by viscometer and potentiometric titration, respectively. The MW was 206.4 kg.mol^-1^ and the DD was 79%. The essential oils (OEs) studied were obtained from Ferquima Ind. e Com. Ltda (Vargem Grande Paulista, São Paulo, Brazil). The lemongrass essential oil (*Cymbopogon flexuosus*; LEO) was obtained from the steam distillation of leaves and the geranium essential oil (*Pelargonium graveolens*; GEO) from the steam distillation of flowers. The LEO and GEO compositions were previously analyzed by gas chromatography, combined with mass spectrometry (GC/MS). A Rtx-5MS column (30 m×0.25 mm×0.25 μm) was briefly used, with column temperature varying from 35 to 180°C at a rate of 4°C/min, then by 180 to 280°C at a rate of 17°C/min, then held at 280°C for 10 min. Helium was used as carrier gas (24.2 mL/min) at the injector temperature of 250°C. The chemical compounds were identified by their relative retention times by gas chromatography and by their mass spectra comparison with NIST database. The main components were found to be citral (83.17%) and citronellol (24.53%).^14^

Chitosan microparticles loaded with LEO and GEO were obtained by spray-drying. Chitosan solution was prepared by dissolving the polymer in acetic acid (0.5% v/v) by mechanical stirring for 24 h. The essential oil was then added to the chitosan solution at a ratio of 10:1 (polymer mass to oil).^14^ The mixture was homogenized at 10,000 rpm for one hour using an Ultra-Turrax 33 homogenizer (IKA Works, Guangzhou, China), followed by sonication for 2 min. The final solution was spray-dried with a Buchi 290 mini spray dryer (Buchi, Flawil, Switzerland) under magnetic stirring until the end of the drying process.

The chitosan microparticles incorporated with essential oils (CM-EOs) used in this study were previously characterized in terms of encapsulation efficiency, process yield, scanning electron microscopy, size, zeta potential, thermogravimetric analysis, FTIR and oil release profiles.^14^ The methodologies and discussions of CM-EOs characterization are summarized in Table 1.

**Table 1:** Essential oil encapsulation efficiency (%EE), zeta potential (mV) values and sizes of chitosan and microparticles

Microparticles	EE (%)	Zeta potential	Size (µm)		
			Max	Min	Mean
Chitosan power	-	+19.02 ± 0.25	-	-	-
Chitosan microparticle	-	+31.87 ± 0.63	14.439	2.194	5.611
CMLEO* 10:1	12.55 ± 0.06	+30.75 ± 0.39	13.315	1.858	4.959
CMGEO** 10:1	35.22 ± 0.02	+46.54 ± 0.69	15.559	1.602	5.009

Note: * Chitosan microparticle loaded with lemongrass essential oil; ** Chitosan microparticle loaded with geranium essential oil

### Microorganisms

The standard strains of *Streptococcus mutans* ATCC 25175 and *Candida albicans* ATCC 10231 were used. Four clinical isolates collected from human mucosa were also employed: *C. albicans* CEMM 03-03-091; *C. albicans* CEMM 03-03-095; *C. albicans* CEMM 03-03-094; and *C. albicans* CEMM 03-03-096. Before each experiment, isolated colonies from strains grown in Mitis Salivarius Bacitracin agar (Sigma-Aldrich, Germany) (*S. mutans*) and Sabouraud agar (Sigma-Aldrich, Germany) (*C. albicans*) were streaked and incubated at 37ºC for 48 h in a CO_2_ atmosphere (5%).

### Susceptibility of planktonic cells

The minimum inhibitory concentration (MIC) of chitosan, essential oils, lemongrass essential oil-loaded (CMLEO) and geranium essential oil-loaded (CMGEO) against *C. albicans* and *S. mutans* planktonic cells were determined by the broth microdilution method, as described in M27-A3 and M7-A7, respectively.^17,18^ All tested compounds were investigated in a concentration range of 2–2048 μg/mL. The MICs were defined as the lowest concentrations able to inhibit 100% of microbial growth compared to the drug-free control well.

The minimum fungicidal concentration (MFC) and the minimum bactericidal concentration of the compounds were determined by plating the wells’ content visible growth on potato dextrose agar, for *C. albicans*, or Mitis Salivarius Bacitracin agar, for *S mutans*. MFC and MBC defined as the lowest drug concentration able to kill 99.9% of microbial inoculum.^19,20^

### Formation of dual-species biofilms

For the mixed species biofilm formation, the inoculation was made in BHI broth (Sigma-Aldrich, Germany) and adjusted to densities of 10^8^ CFU mL^-1^ of *S. mutans* and 10^6^ CFU/mL^-1^ of *C. albicans.*^21^ Each adjusted inoculum (100 µL) was pipetted into a 96-well flat-bottomed microdilution plate. The plate was then incubated at 37ºC for 48 h to allow biofilm growth. Minimum Biofilm Inhibitory Concentration (MBIC) was defined as the lowest concentration capable of causing 50% (MBIC50) and 80% (MBIC80) reduction in biofilm metabolic activity when compared with the drug-free growth control.^22^

### Susceptibility of dual-species biofilms

After biofilm formation, the plate wells were washed twice with sterile PBS and 200 µL of either Chitosan, essential oils, lemongrass essential oil-loaded (CMLEO) and geranium essential oil-loaded (CMGEO), all of them diluted in BHI broth, then tested in concentrations ranging from 256 to 4,096 µg/mL, employing serial dilution.^23^ The plates were then incubated again under the same conditions described above for additional 24 h.^24^

The molecules’ antimicrobial effect was evaluated using the 3-(4.5-dimethylthiazol-2yl)-2.5-diphenyl tetrazolium (MTT) (Sigma-Aldrich, USA)^25^ bromide reduction assay to assess metabolic activity. The microplate wells were washed with PBS, and 125 µL of MTT solution (0.5 mg mL-1) solubilized in sterile PBS with 2% glucose were added. Then, the plates were incubated for 5 h in the dark at 35ºC. After this period, the solutions were removed and 150 µL 100% DMSO were added to each well for 45 min to extract formazan salt. After extraction, 100 µL of the supernatant were transferred to each well of a new microtiter plate and read with a spectrophotometer at 540 nm.

### Scanning electron microscopy (SEM)

To make their architectural differences visible, untreated and chitosan-treated mixed biofilms were evaluated by scanning electron microscopy (SEM),^26^ with minor modifications. Biofilms were formed directly on Thermanox™ coverslips using 12-well tissue culture plates. After a 72-hour growth, the coverslips were washed with PBS and different CM-EO concentrations were added to the samples. Then, they were covered with glutaraldehyde [2.5% in 0.15 M sodium cacodylate buffer (pH 7.2) with 0.1% Alcian blue] and incubated at 25°C for 4 h. The biofilms were then washed twice with cacodylate buffer, and the coverslips were dehydrated in an ascending ethanol series (30, 50, 70, 80, 95 and 100%) for 10 min each, repeating dehydration at the last concentration (100% ethanol). Next, the biofilms were dried at room temperature and covered with hexamethyldisilazane (HMDS) (Polysciences Europe, Germany) for 15 min. Afterward, the HMDS was removed, and the biofilms were dried overnight in a desiccator. Slides were coated with 12 nm of gold (Emitech Q150T, East Sussex, United Kingdom) and analyzed with a Quanta 450-FEG (FEI Company, Oregon, USA) scanning electron microscope in high vacuum mode at 20 kV.

### Cytotoxicity Assay

The samples’ cytotoxicity against the RAW 264.7 cell line was evaluated by the MTT assay.^27^ RAW 264.7 cells were plated (200 µL/well; 96-well plate; 1x10^5^ cells/mL) and incubated for 24 h. The cells were then treated with different concentrations of the compounds. Cells incubated with the vehicle used in the dilution of the samples (supplemented DMEM containing 1% DMSO in the total volume of medium in the well) were used as a positive control group. The cells used as negative control were incubated with 50% DMSO in DMEM (Dx), which is considered a harmful stimulus to cells. After the incubation period, 100 µL of the supernatant were removed and added to 100 µL of MTT solution (1 µg/mL, PBS, pH 7.4). After 4 h of incubation at 37°C in an oven with 5% CO_2_, the supernatant was discarded and 150 µL of DMSO were added to each well to solubilize the formazan crystals. The reading was performed in a microplate reader (Biochrom^®^ Asys UVM340, Cambridge, United Kingdom) under a wavelength of 570 nm, and the data were expressed as a percentage of cell viability using the following formula:


(%)=(×100)÷(

### Statistical analyses

Experimental results were expressed as means ± standard deviations (SD). Student’s t-Test and one-way analysis of variance (ANOVA) were used, followed by the Student-Newman-Keuls test. Differences were considered statistically significant at p<0.05.

## Results

### Susceptibility of planktonic cells

The effects of LEO, GEO, CM, CMLEO and CMGEO on the planktonic susceptibility of *C. albicans* and *S. mutans* are shown in Table 2.

**Table 2:** Minimum inhibitory concentration (MIC), minimum fungicidal concentration (MFC), and minimum bactericidal concentration of chitosan, essential oils and microparticles against *C. albicans* and *S. mutans* planktonic cells

Compounds	*Candida albicans*		*Streptococcus mutans*	
	MIC (µg/mL)	MFC (µg/mL)	MIC (µg/mL)	MBC (µg/mL)
Chitosan	64-512	1,024->2,048	>2,048	>2,048
Chitosan Microparticle	64-512	1,024->2,048	>2,048	>2,048
GEO	512->2,048	512->2,048	128	128
LEO	512-2,048	512->2,048	16	32
CMGEO	8-256	512->2,048	2,048	>2,048
CMLEO	4-128	256->2,048	2,048	2,048

Note: GEO: geranium essential oil; LEO: lemongrass essential oil; CMGEO: chitosan microparticle loaded with geranium essential oil; CMLEO: chitosan microparticle loaded with lemongrass essential oil

All compounds reduced *C. albicans* growth in 100%, with CMGEO and CMLEO showing the best MIC results (8-256 µg/mL and 4-128 µg/mL, respectively), and CMLEO also having the lowest MFC (256 - >2,048 µg/mL) against *C. albicans*. GEO and LEO exhibited the lowest MICs (128 µg/mL and ١٦ µg/mL, respectively) and MBCs (128 µg/mL and 32 µg/mL, respectively) against *S. mutans*.

### Biofilm

**Figure 1:**
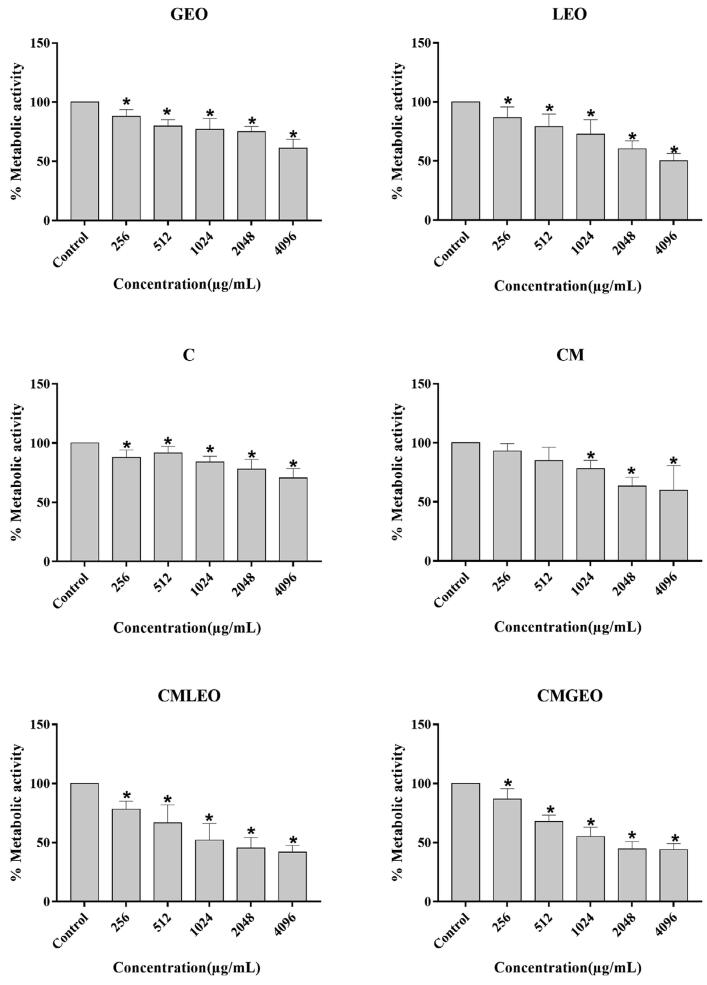
Inhibitory effects of geranium essential oil (GEO), lemongrass essential oil (LEO), chitosan (C), chitosan microparticle (CM) chitosan microparticle loaded with lemongrass essential oil (CMLEO), and chitosan microparticle loaded with geranium essential oil (CMGEO) on the metabolic activity of the mature *C. albicans* and *S mutans* mixed biofilm, compared to mixed biofilms not exposed to the test samples (control). *, *P*˂0.05, the obtained test group values are given as the percentages of biofilm formation in relation to the control group. Results are shown as means ± SD

The effects of LEO, GEO, CM, CMLEO and CMGEO on the metabolic activity of *C. albicans* and *S. mutans* mixed biofilms are shown in Figure 1.

All samples reduced the metabolic activity of *C. albicans* and *S. mutans* mixed biofilms, at all concentrations investigated (p<0.05). The most significant reductions were observed when mixed biofilms were exposed to the highest concentrations of all compounds (p<0.05). LEO and GEO, at the concentration of 4,096 µg/mL (the highest tested concentration), respectively had MBIC50 (Table 3) ranging between 512- >4,096 µg/mL and 2,048-4,096 µg/mL (p<0.05). CMLEO and CMGEO inhibited and reduced the metabolic activity of *C. albicans* and *S. mutans* mixed biofilms more significantly than raw CM and EOs, with MBIC50 ranging between 512-2,048 µg/mL and 1,024-2,048 µg/mL, respectively, suggesting that encapsulation of EOs into CM improved antibiofilm activity.

**Table 3:** Minimum Biofilm Inhibitory Concentration (MBIC) of chitosan, essential oils and microparticles against *C. albican*s and *S. mutans* mixed biofilm

Compounds	MBIC50 (µg/mL)	MBIC80 (µg/mL)
Chitosan	>4096	>4096
Chitosan Microparticle	4096->4096	>4096
GEO	512->4096	>4096
LEO	2048-4096	>4096
CMGEO	512-2048	>4096
CMLEO	1024-2048	>4096

Note: MBIC50 and MBIC80: minimum concentration capable of causing 50% and 80%, respectively, of reduction in biofilm metabolic activity when compared with the drug-free growth control. GEO: geranium essential oil; LEO: lemongrass essential oil; CMGEO: chitosan microparticle loaded with geranium essential oil; CMLEO: chitosan microparticle loaded with lemongrass essential oil

### SEM observation

**Figure 2:**
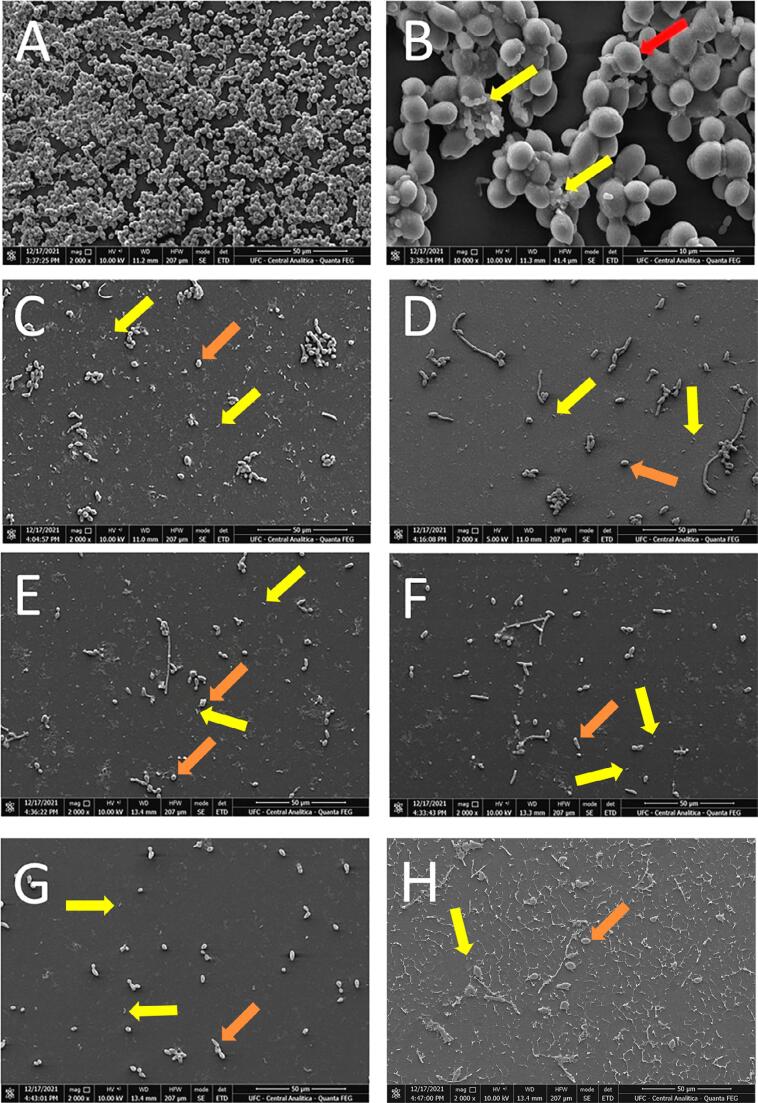
Scanning electron microscopy of two *C. albicans* - *S.mutans* species’ biofilms treated with CM-EOs. Untreated *C. albicans*- *S. mutans* mixed biofilm (A and B), *C. albicans* - *S. mutans* mixed biofilm treated with lemongrass essential oil (C), *C. albicans* - *S. mutans* mixed biofilm treated with 4,096 µg/mL of geranium essential oil (D), *C. albicans* - *S. mutans* mixed biofilm treated with 4,096 µg/mL of chitosan (E), *C. albicans* - *S. mutan*s mixed biofilm treated with 4,096 µg/mL of chitosan microparticle (F), *C. albicans* - *S. mutan*s mixed biofilm treated with 4,096 µg/mL of chitosan microparticle incorporated with lemongrass essential oil (CMLEO) (G), *C. albicans* - *S. mutans* mixed biofilm treated with 4,096 µg/mL of chitosan microparticle incorporated with geranium essential oil (CMGEO) (H). Yellow arrows indicate *S. mutans* cells and red arrows indicate *C. albicans* cells

The duo species biofilm’s architecture was visualized using SEM to investigate the microparticles’ ability to destroy biofilms. Regarding the control, i.e., biofilms that were not treated (Figures 2A and B), a dense population of *S. mutans* and *C. albicans* were observed on the slide surface. Figure 2B shows *S. mutans* colonies attached to *Candida* blastoconidia. In contrast, treatment with 4,096 µg/mL of LEO (Figure 2C), GEO (Figure 2D), Chitosan (Figure 2E), CM (Figure 2F), CMLEO (Figure 2G) and CMGEO (Figure 2H) significantly decreased the number of cells attached to the slide.

### Cytotoxicity assay

**Figure 3:**
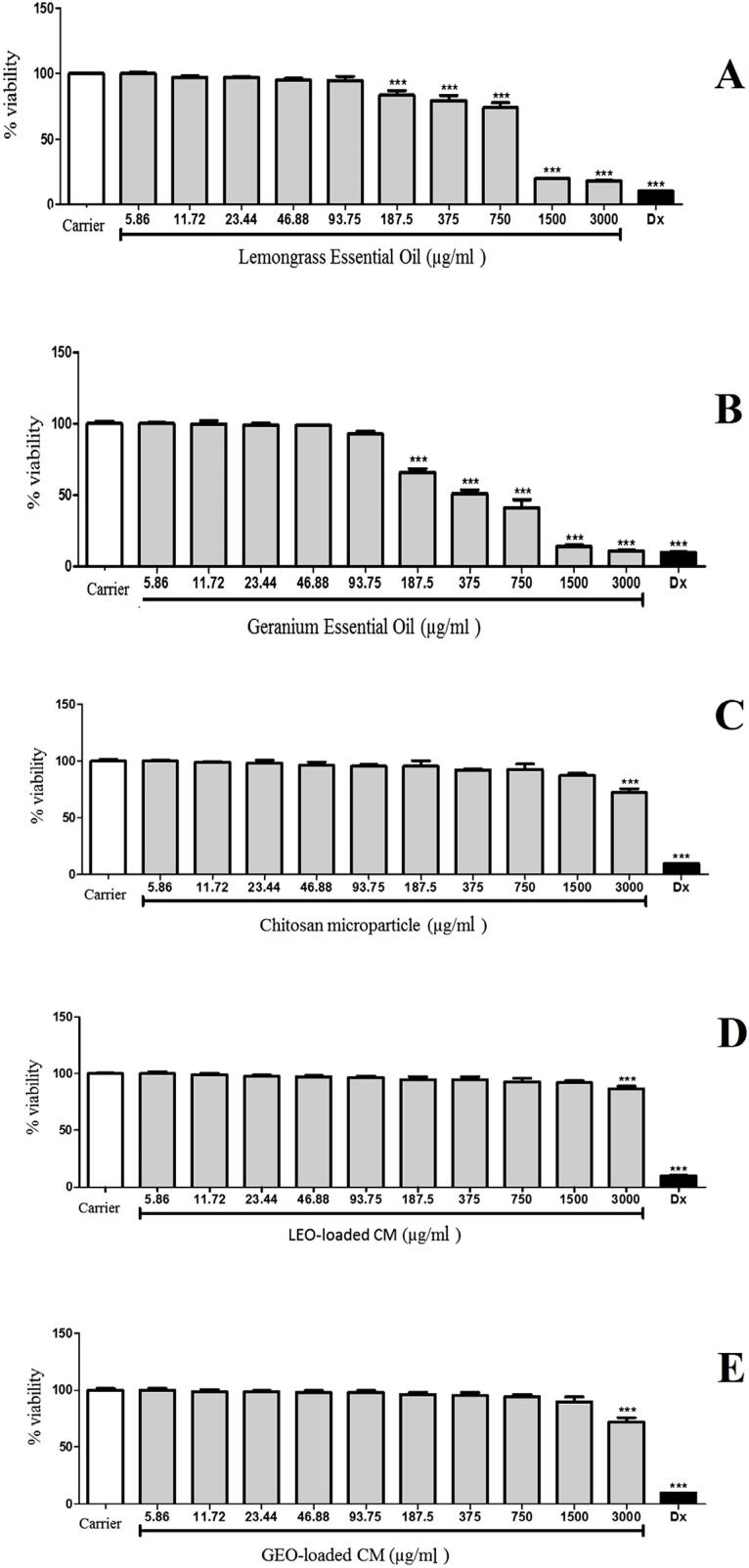
Toxic effects of lemongrass essential oil (A), geranium essential oil (B), chitosan microparticle (C), chitosan microparticle incorporated with lemongrass essential oil (D) and chitosan microparticle incorporated with geranium essential oil (E) on the viability of RAW 264.7 cells. Values were expressed as means ± SEM of three independent determinations (n=8). Vehicle (positive control), Dx = 50% DMSO in medium (negative control). *p<0.05; **p<0.01; ***p<0.001 vs. control (ANOVA, followed by the Student-Newman-Keuls test)

Figure 3 shows the cytotoxic effect of EOs and CM-EOs against RAW 264.7 cells. The MTT assay revealed that CM-EOs were not cytotoxic to RAW 264.7 cells at concentrations ranging from 5.86 to 1,500 µg/mL, as shown by the cell viability greater than 90%. For the CM-EOs, a significant difference (p<0.05) was only observed in relation to the control at a concentration of 3,000 µg/mL. On the other hand, as it can be seen in Figures 2A and 2B, lower cell viability was obtained when RAW 264.7 cells were exposed to essential oils, with a significant difference in relation to the control at low concentrations of oils (187.5 µg/mL).

## Discussion

*C. albicans* and *S. mutans* mixed biofilm is responsible for dental caries, thus being most difficult to eradicate than their mono-species biofilm.^4,28^ Previously, our group showed that chitosan microparticles loaded with geranium (CMGEO) and lemongrass (CMLEO) essential oils had activity against mono-species *C. albicans* biofilms, but the effect of these compounds against mixed biofilm was still unknown.^14^ Herein, the activity of CMGEO and CMLEO against *C. albicans* and *S. mutans* mixed biofilm was tested.

Sensitivity tests showed that chitosan and encapsulated essential oils have better activity against *C. albicans*, whereas essential oils have lower MICs and MBCs against *S. mutans*. As shown in Figure 1 and Table 3, all the samples reduced the metabolic activity of *C. albicans* and *S. mutans* mixed biofilms. Encapsulated oils showed lower MBIC50 than raw chitosan or oils, indicating that encapsulation improved their antibiofilm activity. The activity of chitosan microparticles loaded with geranium (CMGEO) and lemongrass (CMLEO) essential oils against *C. albicans* biofilm had been previously demonstrated – several authors have reported that chitosan and essential oils have antimicrobial activity against various microorganisms.^16,29^ De Paz, et al.^30^ (2011) showed that chitosan nanoparticles had better activity against *S. mutans* biofilms than larger molecules, suggesting that the use of nanoparticles in the encapsulation of EOs could make the system more effective against *C. albicans* and *S. mutans* mixed biofilms.

Duo species biofilms’ architecture was visualized using SEM to investigate the microparticles’ ability to destroy biofilms. Hyphae play an important role in the maturation of *Candida* biofilm.^31^and analysed by microscopy and by qPCR for expression of putative virulence genes. Candida albicans-only biofilms showed limited hyphal production. Hyphal development was significantly (P < 0·001 SEM images demonstrate a mature biofilm with the presence of many hyphae, with *S. mutans* colonies attached to *Candida* blastoconidia and the microparticles reducing the biofilm on the slides. However, the high concentration of CM-EOs required to inhibit biofilms indicates that it would be necessary to optimize them. Carboxymethyl chitosan’s antibiofilm effect on the biofilm of mixed fungi and bacteria species in silicone *in vitro* had been previously demonstrated by SEM assay.^32^ The latter studied work showed that the biofilms treated with carboxymethyl chitosan were less dense with fewer cell layers and morphologically altered cells when compared to the control, which had densely packed cells.

The essential oils showed cytotoxic effects against RAW 264.7 cells at concentrations 8× lower than those that were cytotoxic for CM-EOs, showing a higher cytotoxicity than CM-EOS and justifying their encapsulation into chitosan microparticles. CMLEO showed the lowest cytotoxicity, causing the death of only 30% of the cells even at the highest voluntary concentration (6,000 µg/mL), a value close to that of the chitosan microparticle alone. On the other hand, CMGEO showed higher cytotoxicity, reaching 40%. CM incorporated with isoniazid showed high cytotoxicity values in peritoneal macrophages, but no cytotoxicity in alveolar macrophages.^33^ Thus, future *in vivo* tests are necessary.

The action mechanism of chitosan is based on the electrostatic interaction between the positively charged amino groups and the negatively charged components of membrane and microbial cells’ cell wall. Due to this interaction, chitosan interferes with cell membrane permeability, causing damage and leading to cell death.^34^ In addition, low molecular weight chitosan is associated with another action mechanism, whereby it can penetrate into the cell and interact with mRNA, inhibiting protein synthesis.^35^

To increase antimicrobial chitosan activity, LEO and GEO were encapsulated, which already demonstrated antimicrobial activity against some microorganisms.^36^ A previous study determined LEO composition.^14^ Scientific literature shows that the LEO’s antimicrobial activity happens due to its high content of geranial (α-citral) and neral (β-citral), increasing its biological activity, whereas GEO’s biological activities are associated to its main components, citronellal and geraniol.^37,38^ It has also been reported that, due to EOS’ hydrophobic nature, they can interact with the microbial cell membrane, causing leakage of cytoplasmic content and ions, eventually leading to cell death.^39^ Considering the characteristics of encapsulated oils, positively-charged CM-EOs can bind to negatively-charged biofilm and microbe components, such as sugar polymer and eDNA.^40^ Zeta potential analysis demonstrated that CMLEO and CMGEO have higher positive charges than CM (Table 1). The cationic CM-EOs can penetrate biofilm and bind to the components of anionic biofilm on the surface of microbial cells, acting directly on the internal cells. This could explain how oils and chitosan particles act synergistically.

## Conclusion

CM-EOs and OEs can reduce metabolic activities of duo-biofilms formed by *C. albicans* and *S. mutans*, with encapsulation reducing EOs cytotoxicity. The use of nanoparticles in encapsulation can be an alternative to improve the efficiency of the system against *S. mutans*, but future *in vivo* studies are necessary for a better understanding of the compounds’ toxicity and to optimize its activity by reducing the concentration needed to inhibit the biofilm.

## References

[1] Vila T, Sultan AS, Montelongo-Jauregui D, Jabra-Rizk MA (2020). Oral candidiasis: a disease of opportunity. J Fungi.

[2] Patel M (2022). Oral cavity and Candida albicans: colonisation to the development of infection. Pathogens.

[3] Metwalli KH, Khan SA, Krom BP, Jabra-Rizk MA (2013). Streptococcus mutans, Candida albicans, and the human mouth: a sticky situation. PLoS Pathog.

[4] Garcia BA, Acosta NC, Tomar SL, Roesch LF, Lemos JA, Mugayar LR (2021). Association of Candida albicans and Cbp+ Streptococcus mutans with early childhood caries recurrence. Sci Rep.

[5] Fakhruddin KS, Samaranayake LP, Egusa H, Chi Ngo H, Panduwawala C, Venkatachalam T (2020). Candida biome of severe early childhood caries (S-ECC) and its cariogenic virulence traits. J Oral Microbiol.

[6] Rodrigues ME, Gomes F, Rodrigues CF (2019). Candida spp./Bacteria Mixed Biofilms. J Fungi.

[7] Costa EM, Silva S, Vicente S, Veiga M, Tavaria F, Pintado MM (2017). Chitosan as an effective inhibitor of multidrug resistant Acinetobacter baumannii. Carbohydr Polym.

[8] Garcia LG, Guedes GM, Silva ML, Castelo-Branco DS, Sidrim JJ, Cordeiro RA (2018). Effect of the molecular weight of chitosan on its antifungal activity against Candida spp. in planktonic cells and biofilm. Carbohydr Polym.

[9] Ke CL, Deng FS, Chuang CY, Lin CH (2021). Antimicrobial actions and applications of chitosan. Polymers (Basel).

[10] Ikono R, Vibriani A, Wibowo I, Saputro KE, Muliawan W, Bachtiar BM (2019). Nanochitosan antimicrobial activity against Streptococcus mutans and Candida albicans dual-species biofilms. BMC Res Notes.

[11] Ahmed F, Prashanth S, Sindhu K, Nayak A, Chaturvedi S (2019). Antimicrobial efficacy of nanosilver and chitosan against Streptococcus mutans, as an ingredient of toothpaste formulation: an in vitro study. J Indian Soc Pedod Prev Dent.

[12] Gündel SS, Godoi SN, Santos RC, Silva JT, Leite LB, Amaral AC (2020). In vivo antifungal activity of nanoemulsions containing eucalyptus or lemongrass essential oils in murine model of vulvovaginal candidiasis. J Drug Deliv Sci Technol.

[13] Cortés-Camargo S, Cruz-Olivares J, Barragán-Huerta BE, Dublán-García O, Román-Guerrero A, Pérez-Alonso C (2017). Microencapsulation by spray drying of lemon essential oil: evaluation of mixtures of mesquite gum - nopal mucilage as new wall materials. J Microencapsul.

[14] Garcia LG, Rocha MG, Lima LR, Cunha AP, Oliveira JS, Andrade AR (2021). Essential oils encapsulated in chitosan microparticles against Candida albicans biofilms. Int J Biol Macromol.

[15] Rubini D, Vedha Hari BN, Nithyanand P (2021). Chitosan coated catheters alleviates mixed species biofilms of Staphylococcus epidermidis and Candida albicans. Carbohydr Polym.

[16] Garcia LG, Guedes GM, Fonseca XM, Pereira WA, Castelo-Branco DSCM, Sidrim JJ (2020). Antifungal activity of different molecular weight chitosans against planktonic cells and biofilm of Sporothrix brasiliensis. Int J Biol Macromol.

[17] CLSI (2008). Reference method for broth dilution antifungal susceptibility testing of yeasts; approved standard – third edition.

[18] CLSI (2006). Methods for dilution antimicrobial susceptibility tests for bacteria that grow aerobically.

[19] Scordino F, Pernice I, Passo CL, Galbo R, Medici MA, Criseo G (2015). Antifungal susceptibilities of species of the Sporothrix schenckii complex isolated in Italy. J Biol Res.

[20] Ahrari F, Eslami N, Rajabi O, Ghazvini K, Barati S (2015). The antimicrobial sensitivity of Streptococcus mutans and Streptococcus sangius to colloidal solutions of different nanoparticles applied as mouthwashes. Dent Res J (Isfahan).

[21] Panariello BH, Klein MI, Pavarina AC, Duarte S (2017). Inactivation of genes TEC1 and EFG1 in Candida albicans influences extracellular matrix composition and biofilm morphology. J Oral Microbiol.

[22] Brilhante RS, Pereira VS, Oliveira JS, Lopes RG, Rodrigues AM, Camargo ZP (2018). Pentamidine inhibits the growth of Sporothrix schenckii complex and exhibits synergism with antifungal agents. Future Microbiol.

[23] Karygianni L, Attin T, Thurnheer T (2020). Combined DNase and proteinase treatment interferes with composition and structural integrity of multispecies oral biofilms. J Clin Med.

[24] Martins M, Uppuluri P, Thomas DP, Cleary IA, Henriques M, Lopez-Ribot JL (2010). Presence of extracellular DNA in the Candida albicans biofilm matrix and its contribution to biofilms. Mycopathologia.

[25] Reddy GK, Nancharaiah YV (2020). Alkylimidazolium ionic liquids as antifungal alternatives: antibiofilm activity against Candida albicans and underlying mechanism of action. Front Microbiol.

[26] Brilhante RS, Fonseca XM, Pereira VS, Araújo GS, Oliveira JS, Garcia LG (2020). In vitro inhibitory effect of statins on planktonic cells and biofilms of the Sporothrix schenckii species complex. J Med Microbiol.

[27] Mosmann T (1983). Rapid colorimetric assay for cellular growth and survival: application to proliferation and cytotoxicity assays. J Immunol Methods.

[28] Yang F, Dinis M, Haghighi F, He X, Shi W, Tran NC (2022). Oral colonization of Candida albicans and Streptococcus mutans in children with or without fixed orthodontic appliances: a pilot study. J Dent Sci.

[29] Wan J, Zhong S, Schwarz P, Chen B, Rao J (2019). Physical properties, antifungal and mycotoxin inhibitory activities of five essential oil nanoemulsions: impact of oil compositions and processing parameters. Food Chem.

[30] Paz LE, Resin A, Howard KA, Sutherland DS, Wejse PL (2011). Antimicrobial effect of chitosan nanoparticles on Streptococcus mutans biofilms. Appl Environ Microbiol.

[31] Morse DJ, Wilson MJ, Wei X, Bradshaw DJ, Lewis MA, Williams DW (2019). Modulation of Candida albicans virulence in in vitro biofilms by oral bacteria. Lett Appl Microbiol.

[32] Tan Y, Leonhard M, Moser D, Ma S, Schneider-Stickler B (2016). Inhibition of mixed fungal and bacterial biofilms on silicone by carboxymethyl chitosan. Colloids Surfaces B Biointerfaces.

[33] Oliveira PM, Matos BN, Pereira PA, Gratieri T, Faccioli LH, Cunha MS (2017). Microparticles prepared with 50-190 kDa chitosan as promising non-toxic carriers for pulmonary delivery of isoniazid. Carbohydr Polym.

[34] Ma Z, Garrido-Maestu A, Jeong KC (2017). Application, mode of action, and in vivo activity of chitosan and its micro- and nanoparticles as antimicrobial agents: a review. Carbohydr Polym.

[35] Hosseinnejad M, Jafari SM (2016). Evaluation of different factors affecting antimicrobial properties of chitosan. Int J Biol Macromol.

[36] Sahal G, Woerdenbag HJ, Hinrichs WL, Visser A, Tepper PG, Quax WJ (2020). Antifungal and biofilm inhibitory effect of Cymbopogon citratus (lemongrass) essential oil on biofilm forming by Candida tropicalis isolates: an in vitro study. J Ethnopharmacol.

[37] Shawl AS, Kumar T, Chishti N, Shabir S (2006). Cultivation of rose scented geranium (Pelargonium sp.) as a Cash Crop in Kashmir Valley. Asian J Plant Sci.

[38] Trang DT, Hoang TK, Nguyen TT, Cuong PV, Dang NH, Dang HD (2020). Essential Oils of Lemongrass (Cymbopogon citratus Stapf) induces apoptosis and cell cycle arrest in A549 lung cancer cells. Biomed Res Int.

[39] Feyzioglu GC, Tornuk F (2016). Development of chitosan nanoparticles loaded with summer savory (Satureja hortensis L.) essential oil for antimicrobial and antioxidant delivery applications. LWT.

[40] Ong TH, Chitra E, Ramamurthy S, Ling CC, Ambu SP, Davamani F (2019). Cationic chitosan-propolis nanoparticles alter the zeta potential of S. epidermidis, inhibit biofilm formation by modulating gene expression and exhibit synergism with antibiotics. PLoS One.

